# Conservation physiology of marine fishes: state of the art and prospects for policy

**DOI:** 10.1093/conphys/cow046

**Published:** 2016-10-18

**Authors:** David J. McKenzie, Michael Axelsson, Denis Chabot, Guy Claireaux, Steven J. Cooke, Richard A. Corner, Gudrun De Boeck, Paolo Domenici, Pedro M. Guerreiro, Bojan Hamer, Christian Jørgensen, Shaun S. Killen, Sjannie Lefevre, Stefano Marras, Basile Michaelidis, Göran E. Nilsson, Myron A. Peck, Angel Perez-Ruzafa, Adriaan D. Rijnsdorp, Holly A. Shiels, John F. Steffensen, Jon C. Svendsen, Morten B. S. Svendsen, Lorna R. Teal, Jaap van der Meer, Tobias Wang, Jonathan M. Wilson, Rod W. Wilson, Julian D. Metcalfe

**Affiliations:** 1 Centre for Marine Biodiversity Exploitation and Conservation, UMR MARBEC (CNRS, IRD, IFREMER, UM), Place E. Bataillon cc 093, 34095 Montpellier, France; 2 Department of Biological and Environmental Sciences, University of Gothenburg, Medicinaregatan 18, 413 90 Gothenburg, Sweden; 3 Fisheries and Oceans Canada, Institut Maurice-Lamontagne, Mont-Joli, QC, CanadaG5H 3Z4; 4 Université de Bretagne Occidentale, UMR LEMAR, Unité PFOM-ARN, Centre Ifremer de Bretagne, ZI Pointe du Diable. CS 10070, 29280 Plouzané, France; 5 Fish Ecology and Conservation Physiology Laboratory, Department of Biology, Carleton University, Ottawa, ON, CanadaK1S 5B6; 6 Longline Environment Ltd, 88 Wood Street, LondonEC2V 7RS, UK; 7 Systemic Physiological and Ecotoxicological Research (SPHERE), Department of Biology, University of Antwerp, Groenenborgerlaan 171, B-2020 Antwerp, Belgium; 8 CNR–IAMC, Istituto per l'Ambiente Marino Costiero, 09072 Torregrande, Oristano, Italy; 9 CCMAR – Centre for Marine Sciences, Universidade do Algarve, 8005-139 Faro, Portugal; 10 Center for Marine Research, Ruder Boskovic Institute, Giordano Paliaga 5, 52210 Rovinj, Croatia; 11 Department of Biology and Hjort Centre for Marine Ecosystem Dynamics, University of Bergen, 5020 Bergen, Norway; 12 Institute of Biodiversity,Animal Health and Comparative Medicine, College of Medical,Veterinary and Life Sciences, Graham Kerr Building, University of Glasgow, Glasgow G12 8QQ, UK; 13 Department of Biosciences, University of Oslo, PO Box 1066,NO-0316 Oslo,Norway; 14 Laboratory of Animal Physiology, Department of Zoology, School of Biology, Aristotle University of Thessaloniki, Thessaloniki, Greece; 15 Institute for Hydrobiology and Fisheries Science, University of Hamburg, Olbersweg 24, Hamburg 22767, Germany; 16 Department of Ecology and Hydrology, Faculty of Biology, Espinardo, Regional Campus of International Excellence ‘Campus Mare Nostrum’, University of Murcia, Murcia, Spain; 17 IMARES, Institute for Marine Resources and Ecosystem Studies, PO Box 68, 1970 AB IJmuiden, The Netherlands; 18 Core Technology Facility, The University of Manchester, 46 Grafton Street, Manchester M13 9NT, UK; 19 Marine Biological Section, Department of Biology, University of Copenhagen, Strandpromenaden 5, DK-3000 Helsingør, Denmark; 20 Section for Ecosystem-based Marine Management, National Institute of Aquatic Resources (DTU-Aqua), Technical University of Denmark, Jægersborg Allé 1, DK-2920 Charlottenlund, Denmark; 21 Department of Coastal Systems, NIOZ Royal Netherlands Institute for Sea Research and Utrecht University, PO Box 59, 1790 AB Den Burg, Texel, The Netherlands; 22 Department of Zoophysiology, Aarhus University, 8000 Aarhus C, Denmark; 23 Interdisciplinary Centre of Marine and Environmental Research (CIIMAR), University of Porto, 4050-123 Porto, Portugal; 24 Biosciences, College of Life & Environmental Sciences, University of Exeter, ExeterEX4 4QD, UK; 25 Centre for Environment,Fisheries and Aquaculture Science (Cefas), Lowestoft Laboratory, Suffolk NR33 0HT, UK

**Keywords:** Biomarkers, ecological models, fisheries, Fry paradigm, individual variation, telemetry

## Abstract

The state of the art of research on the environmental physiology of marine fishes is reviewed from the perspective of how it can contribute to conservation of biodiversity and fishery resources. A major constraint to application of physiological knowledge for conservation of marine fishes is the limited knowledge base; international collaboration is needed to study the environmental physiology of a wider range of species. Multifactorial field and laboratory studies on biomarkers hold promise to relate ecophysiology directly to habitat quality and population status. The ‘Fry paradigm’ could have broad applications for conservation physiology research if it provides a universal mechanism to link physiological function with ecological performance and population dynamics of fishes, through effects of abiotic conditions on aerobic metabolic scope. The available data indicate, however, that the paradigm is not universal, so further research is required on a wide diversity of species. Fish physiologists should interact closely with researchers developing ecological models, in order to investigate how integrating physiological information improves confidence in projecting effects of global change; for example, with mechanistic models that define habitat suitability based upon potential for aerobic scope or outputs of a dynamic energy budget. One major challenge to upscaling from physiology of individuals to the level of species and communities is incorporating intraspecific variation, which could be a crucial component of species’ resilience to global change. Understanding what fishes do in the wild is also a challenge, but techniques of biotelemetry and biologging are providing novel information towards effective conservation. Overall, fish physiologists must strive to render research outputs more applicable to management and decision-making. There are various potential avenues for information flow, in the shorter term directly through biomarker studies and in the longer term by collaborating with modellers and fishery biologists.

## Introduction

Marine ecosystems provide essential resources and services, with fishes being of prime socio-economic importance. There are alarming global trends of excessive exploitation and habitat degradation of marine fishes, with most commercial stocks either overfished or nearing capacity (Pauly *et al*., 2002; FAO, 2014; Pauly and Zeller, 2016). Global climate change is also altering patterns of marine biodiversity, with more pronounced effects expected in the future (Perry *et al*., 2005; IPCC, 2014; McNeil and Sasse, 2016). The consequences of over-exploitation, habitat degradation and global climate change are unknown, but there is legitimate concern about irreversible loss of fisheries resources and biodiversity, leading to reduced food security and even direct threats to ecosystem integrity (Perry *et al*., 2005; Cheung *et al*., 2009; Sumaila *et al*., 2011; Duarte, 2014; Elliott *et al*., 2015; Pauly and Zeller, 2016). There is a need, therefore, to improve the scientific knowledge base underpinning advice on the sustainable management of marine fish biodiversity and fisheries resources.

**Figure 1:**
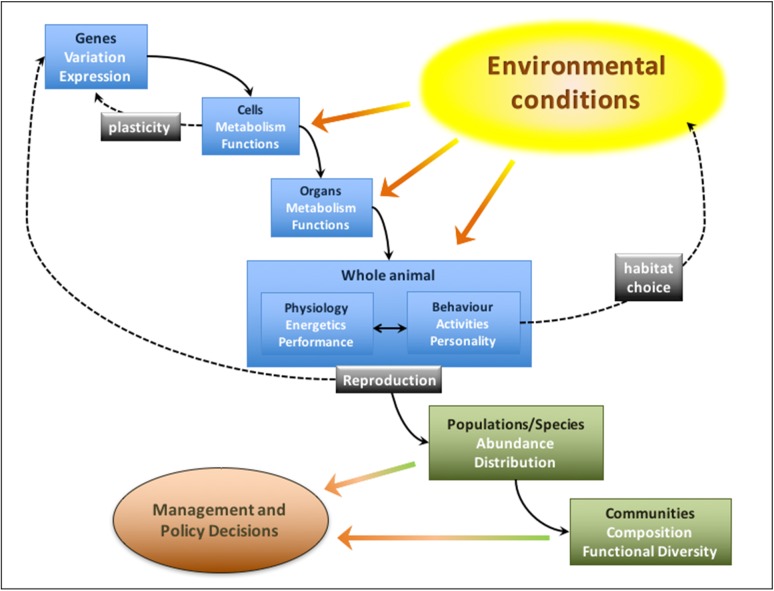
How the physiology of an individual marine fish can influence species population dynamics and community biodiversity, through hierarchical levels of biological organization (inspired by Le Quesne and Pinnegar, 2012). Environmental conditions influence whole-animal physiology (energetics and performance) by influencing gene expression and the biochemistry and physiology (metabolism and function) of cells and organs. Physiology has a complex interplay with the behaviour (activities and personality) of the individual that, together, influence its ecological performance and reproductive output (fitness). This, then, influences the abundance and distribution of the species in the environment. This, in turn, influences the composition and functional diversity of communities and assemblages in the ecosystem. This is the scale at which management decisions are required and made. The dotted black lines show feedbacks. The effects of environmental conditions on cells feed back to influence gene expression, which then influences function by phenotypic plasticity/flexibility. Behavioural habitat choice feeds back to influence environmental conditions and, therefore, their effects on lower levels of organization. Reproduction each generation feeds back to influence genetic variation and drive the evolution of local adaptation in the species.

There is a growing belief that physiological information, understanding how animals function, can contribute significantly to the resolution of management and conservation problems for marine fishes and to the ability accurately to project potential impacts of environmental pressures (Wang and Overgaard, 2007; Pörtner and Farrell, 2008; Rijnsdorp *et al*., 2009; Wilson *et al*., 2009; Pörtner and Peck, 2010; Jørgensen *et al*., 2012; Seebacher and Franklin, 2012; Wilson, 2014). Physiologists typically take a Darwinian view (Fig. 1), whereby the abiotic factors within a given habitat can define which animals survive and reproduce there, based upon their physiology. Over the course of generations, there is natural selection of physiological adaptations to prevailing conditions (Prosser, 1950; Schmidt-Neilsen, 1982; Garland and Carter, 1994), with the evolution of a functional niche for each species (Hutchinson, 1957). In each new generation, the physiology of the individuals contributes to their performance, behaviour and fitness in a realized niche (Arnold, 1983; Garland and Carter, 1994; Feder *et al*., 2000; Huey *et al*., 2012). This influences the abundance and distribution of their population and species (Buckley *et al*., 2012; Huey *et al*., 2012; Chave, 2013; Heffernan *et al*., 2014) and, by logical extension, the composition of communities and assemblages in the ecosystem (Buckley *et al*., 2012; Chave, 2013; Cooke *et al*., 2014; Heffernan *et al*., 2014).

The vast majority of marine fishes are water-breathing ectotherms; therefore, physiological and behavioural responses to increasing temperatures, growing zones of hypoxia, ocean acidification, eutrophication and general habitat degradation are to be expected. This has obvious implications for conservation research because the prediction would be that, as environmental conditions change, so will the distribution of habitat that any given species chooses to, or is able to, occupy (Buckley *et al*., 2012; Huey *et al*., 2012; Le Quesne and Pinnegar, 2012; Cooke *et al*., 2014; Martin *et al*., 2015).

This overall premise was the impetus to establish a network of interested scientists, funded by the European Union's Cooperation in Science and Technology (COST) programme. The COST Action FA1004 ‘Conservation Physiology of Marine Fishes’ (2011–2015) provided a forum for dialogue on this topic, through a series of international conferences and workshops (http://fish-conservation.nu). This article reviews the main issues that were raised and discussed over COST FA1004’s lifetime, with general perspectives on a broad series of topics. These topics fall into four main themes: (i) the state of basic physiological knowledge for marine fishes and how this might be applied directly towards conservation goals; (ii) how physiological knowledge can be integrated into ecological models; (iii) how biotelemetry and biologging studies can contribute to conservation research; and (iv) how conservation physiology research might be rendered relevant and available to decision-makers. These perspectives set the stage for this Theme Section, which comprises reviews, perspectives and research articles that, together, provide an indication of the state of the art of thinking and research in the field.

## The physiological knowledge base is limited

The restricted knowledge base is currently a major constraint to the use of physiology for conservation of marine fishes. Although there are >30 000 species of fishes, knowledge on marine fishes is confined to tens of species, which occur in countries with developed fish ecophysiology research communities. Within these countries, there is a focus on species that are economically or ecologically important and/or are relatively easy to obtain and maintain in captivity. These include temperate species, such as Atlantic cod (*Gadus morhua*), Atlantic salmon (*Salmo salar*), Dover sole (*Solea solea*), European sea bass (*Dicentrarchus labrax*), Pacific salmonids of the genus *Oncorhynchus* or turbot (*Scopthalmus maximus*), plus various tropical species from the Great Barrier Reef. Recent meta-analyses examining how the physiology of ectotherms might shape responses to global change all note the lack of physiological information on a vast majority of fish species and geographical areas (Sunday *et al*., 2012; Seebacher *et al*., 2015; Lefevre, 2016; Killen *et al*., 2016b).

It is essential that physiologists pursue cooperative research projects progressively to fill knowledge gaps (Cattano et al., 2016; Ferreira-Martins, 2016; di Santo *et al*., 2016), to investigate the ecophysiology of many more species, which may be harder to obtain and/or keep in captivity. Focus could be on key elements in food chains and species with specific or rare (Mouillot *et al*., 2013) ecological functions. Studies are needed to evaluate ranges for tolerance of major environmental factors, such as temperature, dissolved gases (hypoxia and hypercarbia), acidification and salinity, within which different marine fish species function effectively, and the thresholds beyond which performance is impaired and survival or reproduction is at risk.

Researchers must keep in mind the complexities inherent to the physiology of marine fishes. For example, the environmental physiology of populations can vary markedly across a species’ geographical range (Conover *et al*., 2006; Gardiner *et al*., 2010), and specific life stages can be critical bottlenecks for population or species persistence in the presence of ongoing global change (Petitgas *et al*., 2013; Ferreira-Martins, 2016). Physiological research on minute marine fish embryos and larvae is technically very challenging (Peck and Moyano, 2016), but these life stages may be the most sensitive to environmental stressors (Killen *et al*., 2007). This Theme Section has two research articles on the ecophysiology of early life stages, with a study of tolerance of little skate (*Leucoraja erinacea*) embryos to hypoxia (di Santo *et al*., 2016) and a study of tolerance of larvae of the ocellated wrasse (*Symphodus ocellatus*) to natural acidification at Mediterranean volcanic seeps (Cattano *et al*., 2016).

A database is currently being prepared for the public domain, as an output of Action FA1004. At present, it comprises effects of temperature on aerobic metabolic scope, digestive energetics (specific dynamic action) and growth, as well as an analysis of hypoxia tolerance, in marine and euryhaline fish species. In this Theme Section, Rogers *et al*. (2016) analyse the database of hypoxia tolerance, measured by respirometry as the critical oxygen partial pressure for regulation of aerobic metabolism (*P*_crit_). This revealed that *P*_crit_ was, as expected, highly variable among species but was also influenced by temperature, CO_2_, acidification, toxic metals and feeding, as well as by the method used to measure it, especially if CO_2_ accumulated in the respirometry system. The database will provide an open repository for a progressive accumulation of physiological trait data, which can be used towards conservation objectives; for example, directly in terms of tolerance thresholds that can be ‘biomarkers’ of environmental stress or to parameterize ecological models, as described below.

## Applications for physiological biomarkers

Biomarkers of environmental pressures hold promise for conservation research in marine fishes (Cooke and O'Connor, 2010); for example, to evaluate ecological quality of habitats within the context of the EU Water Framework Directive or to establish the ‘health’ of populations in particular habitats of interest. A prime example of an endocrine biomarker is plasma vitellogenin, which, in freshwater fishes, has been established as a key indicator of exposure to endocrine-disrupting chemicals (Sumpter and Jobling, 1995; Tyler *et al*., 1996).

If endocrine, cellular and molecular biomarkers are to be useful for conservation research, it is important to understand their limitations, which can include a lack of mechanistic basis for their interpretation, complicated response patterns in wild animals and unclear links to Darwinian fitness (Forbes *et al*., 2006; McKenzie *et al*., 2007; Dantzer *et al*., 2014). The most promising approaches are multifactorial and use a combination of indicators at different levels of biological organization. These allow relationships to be established among measures of functional integrity, such as condition factor or otolith growth rates, and the endocrine, cellular and molecular biomarkers. This, in turn, can then be related to differences in the biotic and abiotic quality of habitats (Cooke and Suski, 2008; Adams and Ham, 2011; Jeffrey *et al*., 2015; King *et al*., 2016; Madliger *et al*., 2016). In developing such suites of biomarkers for conservation research, the focus should be on reliable and user-friendly measures that combine field and experimental approaches and provide ecological relevance (Adams and Ham, 2011; Jeffrey *et al*., 2015; Madliger *et al*., 2016).

Revealing generalized ‘stress’ in natural populations can be very informative. The stress hormones, glucocorticoids, measured in feathers, hair, moulted skin or scat, are widely used in conservation physiology research in wild tetrapods (Dantzer *et al*., 2014). There is evidence that scales can be used in this manner in fishes (Aerts *et al*., 2015), which opens up this practice for conservation research. Cortisol can be measured in fish eggs as a biomarker of maternal stress levels; in tropical reef fishes, increased egg cortisol was linked to poor reproductive success and reduced offspring size (Gagliano and McCormick, 2009). A blood sample can also provide a wealth of biomarker information in fishes. A major problem with wild marine fishes is accounting for the acute stress of capture, but various biomarkers are presumably not sensitive to this, such as some oxidative stress indicators, stress proteins and the expression of stress-related genes in nucleated teleost red blood cells (Beaulieu and Costantini, 2014; Chadwick *et al*., 2015; Jeffrey *et al*., 2015; Madliger *et al*., 2016).

Simple measures of condition factor and energy reserves are informative physiological biomarkers of population health (Claireaux *et al*., 2004; Jeffrey *et al*., 2015) that, in this Theme Section, are applied as biomarkers for effects of parasitism in a small pelagic species, the European anchovy (*Engraulis encrasicolus*), in the northwest Mediterranean (Ferrer-Maza *et al*., 2016). Some studies have measured physiological indicators of whole-animal performance, such as swimming ability, or hypoxia and thermal tolerance, including measurements on fishes in mesocosms or caged at specific sites (Claireaux *et al*., 2004; McKenzie *et al*., 2007; Roze *et al*., 2013). A multifactorial approach has a number of obvious applications in evaluating impacts of major environmental stressors on marine fishes, and to predict the relative sensitivity of different species.

The critical thermal maximum is a physiological ‘biomarker’ of incipient lethal thermal tolerance (Lutterschmidt and Hutchison, 1997), which has been related to ecological phenomena caused by global change (Pörtner and Peck, 2010; Sunday *et al*., 2011, 2012). In natural populations, habitat warming and extreme thermal events can generate sublethal molecular biomarker responses, notably heat shock proteins. Some freshwater Arctic charr (*Salvelinus alpinus*) populations inhabit water bodies that, as a result of global change, now exceed the fish's seasonal thermal optimum. These populations exhibit constitutive heat shock protein and glucose stress responses (Chadwick *et al*., 2015). A history of exposure to extreme warming events can affect glucocorticoid responsiveness to acute stress in coral reef fishes (Mills *et al*., 2015), and the thermal regime during development and incubation may have marked influences on offspring (Zambonino-Infante *et al*., 2013; Moyano *et al*., 2016). There is an opportunity, therefore, to develop databases on lethal thresholds, evaluated as the critical thermal maximum, but also to investigate how evidence of sublethal thermal stress in populations of interest might relate to functional indicators, such as indicators of bioenergetic or nutritional status. Beyond direct management applications, this sort of information would also inform projections of species sensitivity to predicted patterns of global warming.

Loss of equilibrium during progressive hypoxia has been used as an indicator of incipient lethal hypoxic threshold (Anttila *et al*., 2013; Roze *et al*., 2013; Claireaux and Chabot, 2016), and the *P*_crit_ (or O_2crit_) is a physiological ‘biomarker’ of sublethal hypoxia tolerance (Claireaux and Chabot, 2016; Rogers *et al*., 2016). Above the O_2crit_, fishes can show reduced aerobic scope, which can be linked to impairments to physiological performance and reduced appetite. Although these physiological effects of hypoxia may be understood mechanistically, evaluating the impact of environmental hypoxia upon fish ecology and evolution remains difficult in practice. Overcoming this challenge is becoming increasingly important in the face of growing marine hypoxic zones. The constitution of a database gathering key information about species’ oxygen requirements and susceptibility to reduced oxygen availability (Rogers *et al*., 2016) is an important first step. Once again, a multifactorial approach holds promise. Knowledge of hypoxia thresholds, based upon laboratory experiments, could be compared with biochemical, physiological, bioenergetic, nutritional and behavioural indicators in populations of interest, in order to gain insight into the ecological consequences of prevailing hypoxic stress.

Noise pollution can be a major environmental stress for marine fishes, which has been studied for its effects on their behaviour (Slabbekoorn *et al*., 2010; Simpson *et al*., 2016) and their physiology (Nichols *et al*., 2015; Sierra-Flores *et al*., 2015; Celi *et al*., 2016; Simpson *et al*., 2016). In experimental conditions, noise can cause physiological stress responses and upregulation of stress proteins (Nichols *et al*., 2015; Celi *et al*., 2016). Research in aquaculture has shown that Atlantic cod exposed to daily, low-level noise pollution during the spawning window accumulated cortisol in their eggs and had lower egg production and fertilization rates (Sierra-Flores *et al*., 2015). It seems evident, therefore, that further research should be performed to investigate responses to noise pollution in natural populations, applying a multifactorial approach (Adams and Ham, 2011; Jeffrey *et al*., 2015) that could also include maternal effects, such as accumulation of cortisol in eggs and the potential downstream effects on larvae. Research should distinguish between the type and intensity of noise, on a continuum from not detectable to chronic but allowing habituation to acute and damaging.

In Europe, reforms to the Common Fisheries Policy have made discard of unwanted bycatch an important policy issue, and one where physiological biomarkers clearly have useful applications. This is a very active area of research, so this is restricted here to some generalizations about management needs that are useful to highlight for anyone who is starting out, as follows: (i) characterize the relative sensitivity of different species relative to gear types, environmental conditions and handling procedures; (ii) predict mortality (and sublethal fitness impacts) of discarded fishes (Davis, 2010); and (iii) identify strategies for reducing stress, injury and mortality and improving welfare (Metcalfe, 2009). Bearing in mind that all captured fishes will experience some level of stress and injury, these effects are related and, therefore, may be difficult to disentangle. It is difficult to generalize across species and capture method, and impacts may vary seasonally and ontogenetically. Even fishes that escape capture may suffer impacts of some kind.

## Is there a universal paradigm linking physiological function to ecological performance?

**Figure 2:**
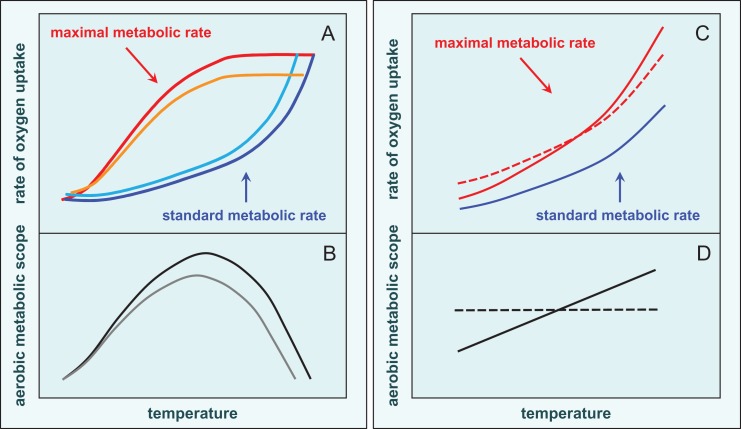
(**A**) The Fry or oxygen- and capacity- limited thermal tolerance (OCLTT) paradigm, a conceptual model of how environmental factors influence aerobic metabolic scope (AS) of fishes (Fry, 1971; Pörtner, 2010). Acclimatization temperature, on the abscissa, is a factor that controls the rates of all metabolic processes in ectothermic marine fishes. The blue and red lines in (A) model how standard (minimal) metabolic rate (SMR) and maximal metabolic rate (MMR) vary as a function of temperature. The difference between SMR and MMR is AS, the capacity of the cardiorespiratory system to provide oxygen for all activities above maintenance, i.e. locomotion, digestion and tissue growth. The SMR, the cost of vital basal processes, increases exponentially with temperature, owing to direct acceleratory effects of heat. At low temperatures, MMR is low because of depressive effects of cold on biochemistry and physiology, including mitochondrial oxygen supply and ATP production. As a result, AS is small. As temperature rises, AS increases as biochemical and physiological rate capacity increase. At a certain temperature, however, MMR reaches an asymptote, attributable to intrinsic limitations to cardiorespiratory capacity. At temperatures above this, SMR rises inexorably until basic maintenance requires the entire cardiorespiratory capacity, so AS falls to zero. (**B**) The resultant relationship of AS with acclimatization temperature, with a clear bell-shaped performance curve and a temperature range where AS is maximal. The form of this curve is expected to be species (or even population) specific and to reflect evolutionary history. The other lines in (A) show effects of loading factors, such as stress (light blue), and limiting factors, such as hypoxia (orange), with the resultant reduction in AS shown in (B) in grey. Figure redrawn from McKenzie (2011). (**C**) The relationship between SMR, MMR and temperature that is shown by many species, with (**D**) showing the resultant relationship of AS with temperature (Lefevre *et al*., 2016). All fishes show a similar effect of temperature on SMR, but some show a parallel increase in MMR, such that scope is the same across all temperatures (dashed MMR and AS lines in C and D, respectively). Other species show a greater increase in MMR than in SMR, such that scope rises progressively with temperature (continuous MMR and AS lines in C and D, respectively).

Understanding the physiological mechanisms that determine how marine fishes perform, in relationship to environmental conditions, should contribute to conservation activities by providing insights into current and future species abundance and distribution (Pörtner and Farrell, 2008; Pörtner, 2010; Jørgensen *et al*., 2012; Teal *et al*., 2015; Marras *et al*., 2015a). One major hypothesis to define how environmental conditions affect performance, with implicit consequences for population dynamics and habitat selection, focuses on the ability of fishes to increase their rate of oxygen uptake to meet the metabolic demands of essential activities. First formulated by F. E. J. Fry, hence called the ‘Fry paradigm’ (Fry, 1971, 1947; Priede, 1985; Kerr, 1990), it is based upon scope for aerobic activity (Fig. 2). Aerobic scope is the integrated capacity of the cardiovascular and respiratory systems to provide oxygen for essential activities beyond vital basal metabolic processes, i.e. activities such as locomotion (e.g. evading predators, foraging, social interactions, migration), digestion and somatic and gonadal growth (Fry, 1971; Priede, 1985; Claireaux and Lefrançois, 2007; Pörtner and Farrell, 2008; Schulte, 2015; Baktoft *et al*., 2016). Thus, the hypothesis provides a mechanistic link from the structural, biochemical and physiological components of metabolism to ecologically relevant performance measures. There is evidence that large-scale failures in upriver spawning migrations of adult Pacific salmon (*Oncorhynchus* species) may have occurred because abnormally high summer temperatures impaired swimming performance through reduced aerobic scope, which is one of the most prominent examples of conservation physiology research for fishes (Eliason *et al*., 2011; Patterson *et al*., 2016).

An important potential strength of the Fry paradigm is that it can integrate effects of major environmental stressors (Fig. 2), notably hypoxia, ocean acidification and pollutants, because they can all constrain aerobic scope (Claireaux and Lagardère, 1999; Lefrançois and Claireaux, 2003; Claireaux and Lefrançois, 2007; Ishimatsu *et al*., 2008; Claireaux and Chabot, 2016). This focus on aerobic metabolism of fishes was the stimulus for a previous journal special issue by COST FA1004, which provided definitions and methods for measures of metabolic rate in fishes, and case studies illustrating the relevance of metabolic rate in management of fishing and environmental changes (Chabot *et al*., 2016).

The Fry paradigm was the basis for the more mechanistic oxygen- and capacity- limited thermal tolerance (OCLTT) hypothesis, which posits that the decline in aerobic scope outside an optimal temperature range is caused by an impaired capacity of mitochondria to use oxygen at low temperatures or of the cardiorespiratory system to supply oxygen at high temperatures (Pörtner and Knust, 2007; Pörtner and Farrell, 2008; Pörtner, 2010; Schulte, 2015). An influential study by Pörtner and Knust (2007) indicated a correlation across laboratory measurements of aerobic scope at various temperatures with thermal tolerance, growth rate and local abundance of a wild population of the eel pout (*Zoarces viviparous*). The Fry and OCLTT paradigms have attracted considerable research attention for fishes (Clark *et al*., 2013; Gräns *et al*., 2014; Norin *et al*., 2014; Wang *et al*., 2014; Brijs *et al*., 2015) and other ectotherms (Overgaard *et al*., 2012; Ern *et al*., 2014; Fobian *et al*., 2014; Verberk *et al*., 2016), especially to investigate the mechanistic tenets of the OCLTT hypothesis. In this Theme Section, Baktoft *et al*. (2016) found no phenotypic covariance between aerobic scope of European perch (*Perca fluviatilis*), as measured in the laboratory, and their spontaneous swimming activity in the wild, suggesting that other factors may override any links between scope and fish performance in routine ‘benign’ conditions (Killen *et al*., 2013). Careful laboratory studies on various fishes have failed to find evidence that aerobic scope declines when they are near their upper lethal temperature or that oxygen delivery is the factor defining tolerance of acute warming (Norin *et al*., 2014; Wang *et al*., 2014; Brijs *et al*., 2015). A study by Claësson *et al*. (2016) on European eel (*Anguilla anguilla*), in this Theme Section, reports that aerobic scope increases with acute warming, underpinned by increases in cardiac output, until temperatures that are almost lethal. Thus, the universality of the Fry and OCLTT paradigms has been questioned, and this remains an active debate (Clark *et al*., 2013; Schulte, 2015; Farrell, 2016).

Also in this Theme Section, Lefevre (2016) presents a comprehensive review and analysis of the effects of temperature and mild hypercarbia (reflecting projected increases in water CO_2_), alone and in combination, on aerobic scope in fishes and other marine ectotherms. This revealed more variation in the response of aerobic scope to elevated temperature and CO_2_ than would be predicted by the Fry and OCLTT paradigms. Although some species exhibited an aerobic performance curve that rose and then declined as a function of increasing temperature, a considerable number of species did not. Some exhibited no change or a decrease in aerobic scope, whereas many exhibited a constant increase, without any mortality, as they were warmed towards their lethal threshold (Fig. 2; Lefevre, 2016). The effects of elevated CO_2_ also varied among species, often being without effect or sometimes increasing aerobic scope. In cases where hypercarbia suppressed aerobic scope, high temperature sometimes had a synergistic effect, but a simple additive effect was the most common (Lefevre, 2016).

Overall, although it is intuitive that physiological energetics will be of ecological significance for aquatic ectotherms (Fry, 1947, 1971; Ware, 1982; Priede, 1985; Kerr, 1990; Jørgensen *et al*., 2012), it would also be unwise blindly to assume that the Fry and OCLTT paradigms hold for all marine fish species (Lefevre, 2016). The effects of temperature on aerobic scope may depend upon a species’ ecology and history of exposure to diurnal or seasonal temperature variations (Norin *et al*. 2014; Lefevre, 2016). A lasting impact of the paradigms, whatever might be learned about their universality, is that they have focused attention on how thermal performance curves can provide a mechanistic link between physiology and ecology for fishes (Schulte, 2015). In particular, they can be integrated into ecological models to provide insights for management and conservation.

## Integrating physiology into ecological models

Models are now important tools for projecting the impact of global change on abundance and distribution of marine fishes. The ability to transfer knowledge of historical observations and make robust projections of future distributions is essential to provide sound advice for management decisions (Jørgensen *et al*., 2012; Cooke *et al*., 2014; Peck *et al*., 2016). This is an area where physiology is perceived to hold great promise for conservation research, through integration into mechanistically based models of habitat suitability, which should provide increased confidence in projections (Hollowed *et al*., 2011; Jørgensen *et al*., 2012, 2016; Teal *et al*., 2012, 2015; Cooke *et al*., 2013, 2014; Deutsch *et al*., 2015; Marras *et al*., 2015a; Peck *et al*., 2016).

Species distribution models are a common ecologically based approach, which use associations between aspects of habitat and known occurrences of species in order to define sets of conditions in which species are likely to occur (Ben Rais Lasram *et al*., 2010; Albouy *et al*., 2012, 2013). The correlative approach has contributed significantly to projections of the potential effects of climate change on marine fish distributions. Its practical advantages are simplicity and flexibility in data requirements, and the range of biotic/abiotic interactions that can be incorporated (Kearney and Porter, 2009). Such correlative approaches are not, however, underpinned by mechanistic causalities, which is a prerequisite for confident projections of species range shifts (Jørgensen *et al*., 2012; Teal *et al*., 2015).

Physiology-based models should be able to deal with these issues of extrapolation because the organismal response is measured in the laboratory in controlled environmental conditions. Furthermore, physiology-based models overcome the circularity of predicting species response to climate change using range filling of potential distributional areas (Teal *et al*., 2015; Peck *et al*., 2016). Models that incorporate physiology typically focus on energetics because of the intuitive link to ecological performance (Jørgensen *et al*., 2012). These models vary in the assumptions and structure of the physiology that is included, from the empirically driven Wisconsin school of bioenergetics modelling (Hewett and Johnson, 1987; Hanson *et al*., 1997) to dynamic energy budget (DEB) models that strive for a universal description of organismal energetics derived from first principles (Kooijman, 1993, 2010). That being said, the integration of physiology into models is an area where further research and input are vitally needed. There is a need for reliable knowledge about how fishes function, in order to ensure that ‘universal’ traits of energetics are valid and are correctly represented in model parameterizations. This is essential to improve confidence in predictions about effects of climate change (Brander, 2015; Peck *et al*., 2016). The debate surrounding the Fry and OCLTT paradigms’ performance curves has already been mentioned, and the physiological principles underlying some other influential model projections (Pauly, 1981; Cheung *et al*., 2011, 2012) have also been questioned (Brander, 2015).

**Figure 3:**
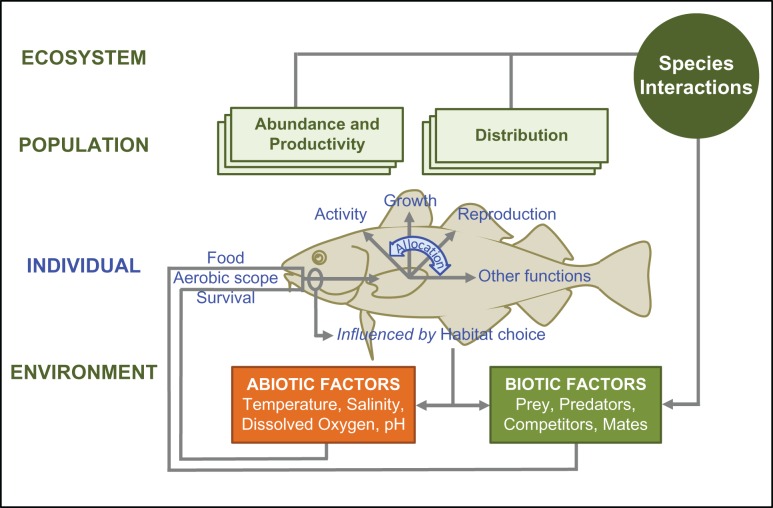
A schematic diagram of how aerobic scope can be integrated into mechanistically based models. It can be used to form a performance curve with temperature, to describe habitat suitability (metabolic maps, e.g. Marras *et al*., 2015a) and it can be a constraint for oxygen allocation to competing activities in life-history models (Holt and Jørgensen, 2014, 2015). See Jørgensen *et al*. (2012) for more details.

**Figure 4:**
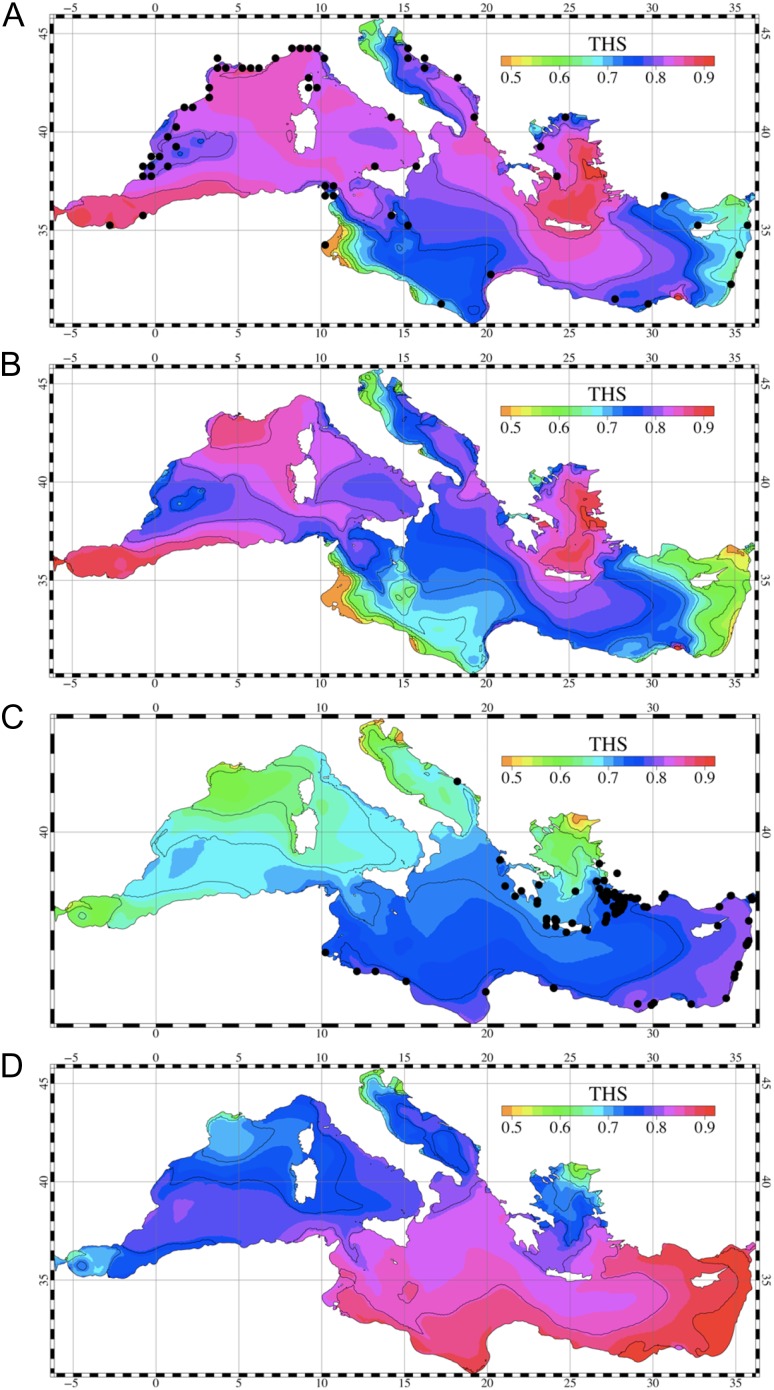
An example of metabolic maps for two herbivorous fishes in the Mediterranean, developed by combining an aerobic scope performance curve with oceanographic data (for details, see Marras *et al*., 2015a). Thermal habitat suitability (THS) was computed for the whole Mediterranean Sea from the basin-scale model results. (**A**) Thermal habitat suitability of a native species, salema (*Salpa salpa*), based on present-day simulation results. (**B**) Thermal habitat suitability of the salema projected for a future scenario. (**C**) Thermal habitat suitability of an invasive lessepsien species, the marbled spinefoot (*Siganus rivulatus*), based on present-day simulation. (**D**) Thermal habitat suitability of the marbled spinefoot projected for the future scenario. Black dots represent the sites where the fish species have been observed.

Aerobic scope can be a useful physiological parameter for models that link individual energetics to processes at higher biological levels (Fig. 3), and which can incorporate interactions among stressors (Jørgensen *et al*., 2012). One approach has used aerobic scope to define habitat suitability, based on laboratory measurements of it as a function of acclimatization temperature in target species, coupled with oceanographic modelling. The model outputs include ‘metabolic maps’ (Del Raye and Weng, 2015; Deutsch *et al*., 2015; Marras *et al*., 2015a,b; see also Martin *et al*., 2015) based on the hypothesis that scope is an indicator of relative fitness potential (Fig. 4). Models based on aerobic scope can be useful in studies of invasive species, by projecting the relative performance of a native species and its competitor counterpart, thus estimating the ‘winners’ and ‘losers’ under climate-driven change for various locations and at different times (Marras *et al*., 2015a). Other applications could include studies on key predators or prey species, in order to evaluate possible effects of global change on trophic relationships and food webs.

There are also life-history models that incorporate aerobic scope as a constraint in life-history evolution, in order to explore its links to fitness (Holt and Jørgensen, 2014, 2015; Jørgensen *et al*., 2016). They integrate the physiology of oxygen uptake and use with foraging and digestion and with life-history traits, such as growth, survival and reproduction (Holt and Jørgensen, 2014, 2015). When these characteristics of an individual were optimized together in a model for Atlantic cod, simulations suggested that fitness would rapidly decline at high temperatures as a result of energy-budgeting conflicts (Holt and Jørgensen, 2014, 2015; Jørgensen *et al*., 2016), driven in part by increased food requirements (Johansen *et al*., 2015). These models are interesting because their projections appear to be relatively robust to the shape of the aerobic scope performance curve near the lethal limit, because fitness peaked at cooler temperatures (Holt and Jørgensen, 2014, 2015; Jørgensen *et al*., 2016). Thus, irrespective of any doubts about the universality of the Fry or OCLTT paradigms, these life-history models suggest that oxygen budgets may well define a main constraint for future projections of marine fishes under environmental change (Holt and Jørgensen, 2014, 2015; Johansen *et al*., 2014, 2016).

**Figure 5:**
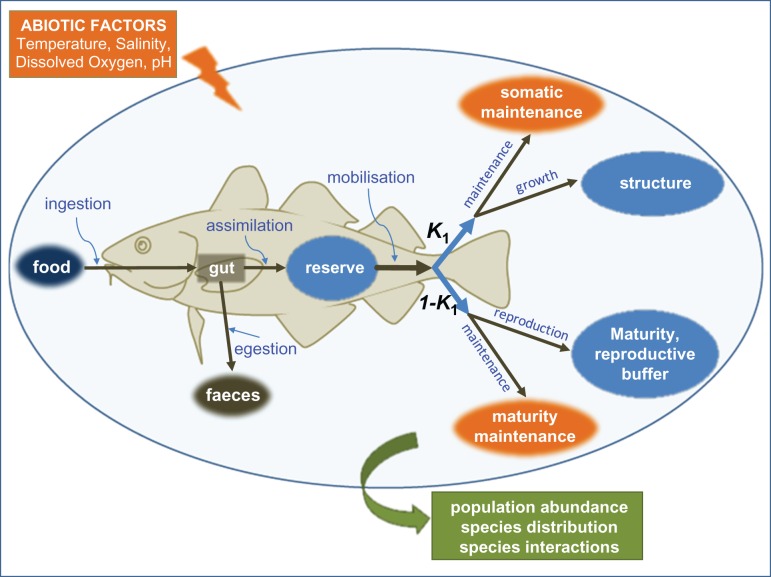
Schematic representation of the standard dynamic energy budget (DEB) model showing the paths of energy flow through a fish (or any organism). Sources or sinks of energy are shown as green, brown and orange ovals; the blue ovals are the three state variables describing the organism. Processes affecting energy flows are indicated by black arrows. A defining feature of DEB models is the existence of reserves, from which allocation rules (proportion *K*_1_) define the partition of energy among processes such as maintenance (somatic or gonadal), somatic growth and reproduction. Dynamic energy budget models can be parameterized to account for effects of abiotic variables, and their universal principles allow for interspecific comparisons of parameter estimates. See Teal *et al*. (2012) for details of an application to evaluate and project marine fish habitat suitability.

Dynamic energy budget theory has also been used as a mechanistic basis to model habitat suitability (Teal *et al*., 2012, 2016; Raab *et al*., 2013). The theory is grounded in the idea that metabolism is organized in the same way within all organisms, including fishes (Fig. 5). It derives from a number of assumptions that can describe empirical patterns, such as the van Bertlanffy growth curve or Kleiber's rule (for a list, see Sousa *et al*., 2008), which are consistent throughout the animal kingdom. The advantage is that the standard DEB model can be applied to all organisms and therefore all fish species, with each described by a set of species-specific parameters. Although parameterization requires empirical data, if data are lacking the model can still provide useful insights with data from related species for which more is known. The potential applications of the DEB-based models are similar to the aerobic scope models, but their particular value is that they provide outputs of growth and fecundity in relationship to environmental conditions, such as temperature or food availability. Dynamic energy budget modelling, in combination with ecosystem models that provide spatial and temporal data on environmental conditions, has been used to develop maps of optimal habitats for growth of marine flatfishes and to project these under climate-driven warming (Teal *et al*., 2012). The DEB theory can also be used to investigate effects on energetic pathways of other stressors, such as hypoxia, acidification or pollutants, if data are available.

Physiological models based on aerobic scope or DEB need to be integrated with physical ocean models and validated against population- and community-level data, so that they can achieve their promise. This would allow them to contribute to, for example, the Intergovenmental Panel on Climate Change (IPCC) predictions for effects of warming on global marine fisheries (Cheung *et al*., 2009, 2013; Sumaila *et al*., 2011). The ability to investigate how other processes, such as hypoxia, ocean acidification and trophic disruption, will interact with warming is now recognized as a research priority (Gunderson *et al*., 2016) and is a major strength of the models that incorporate aerobic scope or DEB. This has been highlighted as an area of great uncertainty in other physiology-based models (Brander, 2015). Once again, however, application of physiology in models requires more information on many more marine fish species. Parameterizing any mechanistically based model with valid physiological data could be a major undertaking, requiring significant long-term studies, use of facilities and personnel. International collaboration and funding are therefore required to coordinate development of laboratory and field measurements of physiology and physiologically based models.

Embedding physiological knowledge of species within models representing the spatial dynamics of marine food webs can provide concrete advice for fish conservation. This is especially true in light of the current emphasis on ecosystem-based fisheries and ecosystem management in Europe and elsewhere. Ecophysiologists and modellers can collaborate to create new tools, beyond well-established models, such as the Ecopath with Ecosim (Christensen and Walters, 2004) or species distribution models based on bioclimate envelopes (Peck *et al*., 2016), to enhance understanding of fishes and their responses to global change and to provide knowledge and tools to support adaptive management (Williams, 2011; Elliott *et al*., 2015; Queirós *et al*., 2016).

## The significance of individual variation

A major challenge for conservation of biodiversity is to understand the capacity of species to acclimatize and, ultimately, to adapt genetically to ongoing global change (Seebacher and Franklin, 2012; Crozier and Hutchings, 2014; Seebacher *et al*., 2015). A core issue is to understand the different facets of intraspecific diversity, i.e. the differences among individuals within a population or species that are the substrate for evolution by natural selection. Of particular interest, from a conservation perspective, is the individual variation in physiological sensitivity to environmental conditions that exists within a given species, including how this may vary among populations across their geographical range, as a result of local adaptation. This variation needs to be understood in itself, as an indication of the potential resilience of a given population or species to environmental change and habitat modification. Ultimately, the goal is to understand how such variation links to life-history variation, to adaptation and evolution of the population or species, and so to underlying heritable genetic variation. Such associations are far from being understood in marine fishes, even for the most intensively studied species, such as Atlantic cod or Atlantic salmon.

The causes and consequences of individual variation in physiology are currently major areas of research, and there are many fundamental and ‘mechanistic’ physiological questions with conservation implications, such as the significance for an ecosystem approach to fisheries (Killen *et al*., 2015; Ward *et al*., 2016). Intraspecific diversity can have a genetic basis but it can also vary with life stages and sex and be affected by transgenerational maternal effects and early life experience (Gore and Burggren, 2012; Ho and Burggren, 2012; Miller *et al*., 2012; Zambonino-Infante *et al*., 2013). One area of individual physiological variation that is of major interest is ‘metabolic phenotypes’, meaning animals with different metabolic rates and aerobic scopes, and the ecological and evolutionary significance of this (Metcalfe *et al*., 2016b). An associated core issue, which transcends physiology, is to understand how major physiological, behavioural and life-history traits might co-vary, whether they might associate into syndromes, and how these might be maintained by ecological trade-offs (Réale *et al*., 2010; Careau and Garland, 2012; Killen *et al*., 2013). These various questions are far too broad and complex to be reviewed adequately here, but they are very poorly understood in marine fishes as a whole. A perspectives paper in this Theme Section considers these issues and, therefore, the reasons why individual variation should be taken into account in the ecosystem approach to fisheries (Ward *et al*., 2016).

Although physiological traits are often attributed ecological and evolutionary significance, there is a need to investigate trait repeatability in wild fish populations and whether the temporal stability of traits may be affected by changing environmental conditions. Temporal stability of physiological traits, plus a genetic component to the observed intraspecific variation, is a prerequisite for a trait to be a target for natural selection. This would influence the ability of species to evolve the trait in response to environmental conditions. Changing environments may erode or enhance trait repeatability, possibly changing which traits are under direct and correlated selection. At present, investigation of potential effects of climate change in marine fishes have primarily examined how warming or ocean acidification can influence population means for variables such as locomotory capacity, metabolism or behaviour (Seebacher *et al*., 2015; Lefevre, 2016; Nagelkerken and Munday, 2016). The current lack of information about how such environmental disturbances affect trait repeatability is a crucial gap that hinders the ability to predict how populations can cope through evolutionary responses. Ongoing advances in respirometry and biotelemetry/biologging, in particular, should increase understanding of trait repeatability in marine fishes and its response to changing environments. The repeatability of traits, and the extent to which this is context dependent, is the topic of a review by Killen *et al*. (2016a) in this Theme Section, with consideration of the implications for management and conservation of fish populations.

It is worthwhile to consider whether ecological models can incorporate individual variation, how these might be parameterized, and whether new models might be needed. Unstructured population models, such as the Lotka–Volterra competition or predator–prey model, assume that all individuals are equal. Age-structured models, such as the well-known Leslie matrix model (Caswell, 2000), take ontogenetic stage into account but ignore other potential sources of variation, assuming a constant and similar environment for all individuals and that animals of the same age remain exactly the same across time. Physiologically structured population models consider the potential for a variable environment to introduce variation, such that animals of a similar age may differ significantly, allowing for richer ecological interactions (de Roos, 1997; de Roos and Persson, 2013). The basic physiology and behaviour of each individual in these models is characterized by a parameter vector, which thus represents the genotype, and the vector of state variables represents the phenotype. The DEB model of the individual can serve as a building block for these physiologically structured models, with an example provided by van der Meer (2016) in this Theme Section. All individuals usually have the same parameter vector and thus the same genetic constitution in physiologically structured population models (Kooi and van der Meer, 2010). Studies to investigate the evolutionary stability of populations require genetic variability. Adaptive dynamics models (Dieckmann and Law, 1996), for example, aim to find a population where mutants (with a slightly different vector to residents) can no longer invade (Metz *et al*., 1992). The approach normally assumes clonal reproduction, but sexual reproduction can be incorporated, such that diversity among sexes can be considered. In other models (e.g. Giske *et al*., 2014), the genotype is explicitly modelled, which allows for emergent genetic variation and coexistence of different genotypes and phenotypes (e.g. behavioural strategies).

Many current population and community models ignore all potential sources of individual variation. Given that there is a huge difference between an Atlantic cod larva and full-grown adult, and a huge difference in growth rate between a well-fed or starved cod, ontogenetic stage and environmental history should, at least, be incorporated into population models. Beyond that, certain phenomena may not be understood and may not be predicted well if such variation is not considered (Ward *et al*., 2016). Furthermore, the variation may itself be of interest and provide insights into the underlying biological processes that have produced and maintained it. Given that parameterizing models to account for the various different potential facets of individual variation is a major long-term undertaking, in terms of facilities and personnel, pragmatic alternatives are worthy of careful investigation. One such alternative is pattern-oriented modelling (Grimm *et al*., 2005), which focuses on empirically quantifying processes and key trade-offs that cause or constrain variation, then using a model to predict individual variation and compare it with observed variation.

## Understanding what fishes do in nature

It remains a central problem to relate the physiology of fishes, measured in the laboratory, to the habitats and conditions they experience (and will select) in their natural environment. There are immense technical difficulties in following fishes in the vast three-dimensional marine realm, let alone in measuring physiological variables or estimating their physiological state and whether they occupy habitats that optimize some element of their physiology (Freitas *et al*., 2016).

Active and passive acoustic tracking is already widely used in marine fishes, in order to follow them and estimate variables such as swimming speed and distance, plus two- and three-dimensional positioning. The acoustic signal carries over only relatively limited spatial scales, but the ongoing development of networks of acoustic receivers along coastlines, such as the Ocean Tracking Network (http://oceantrackingnetwork.org/research/canadian-projects/), will provide extremely valuable information about, for example, habitat use or migration patterns of marine fishes, that has major applications for conservation research and policy. Rapidly evolving techniques of measurement of physiological variables from free-living animals, including fishes, have been suggested to provide ‘answers to questions that we did not know we should ask’.

**Figure 6:**
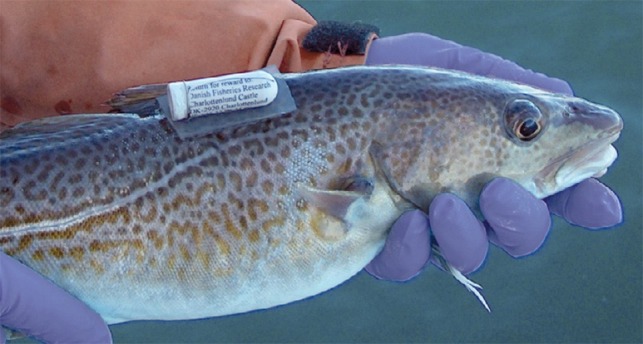
An Atlantic cod (*Gadus morhua*) carrying a data storage tag that records pressure, temperature and salinity. Photograph: Stefan Neuenfeldt, DTU Aqua.

The first biotelemetric measurement was probably performed by Marey (1896). Biotelemetry and biologging (Fig. 6) are now starting to provide information on the physiology of animals in the field. Together with tracking data, they are providing a better picture of the life cycles of some economically important species, plus information about the structure of their populations (Metcalfe, 2006; Rutz and Hays, 2009; Block *et al*., 2011; Metcalfe *et al*., 2012; Whitlock *et al*., 2015). These tools can record an animal's physiology while simultaneously recording environmental conditions around it, in order to investigate assumptions based on laboratory experiments. For example, some acoustic telemetry tags can measure oxygen content in the water surrounding a fish and transmit this in real time (Svendsen *et al*., 2006). There are emerging techniques to collect physiological information on free-swimming fishes, which can then be used to estimate energetics as a function of prevailing environmental conditions (Gräns *et al*., 2010; Wright *et al*., 2014; Metcalfe *et al*., 2016a). Third-generation biotelemetry systems are being developed for simultaneous measurement of multiple physiological variables; for example, blood flow, blood pressure, electrocardiograms, electromyograms, three-dimensional acceleration and temperature. These can have a bidirectional radio frequency link that allows the implant to send data and accept commands to perform tasks. The signal from the implant can be viewed online, with a transmission range of ~10 m in air. This is, however, reduced in water, especially sea water, where alternative strategies are required, such as acoustic signalling or biologging.

Biologging, where the physiological data are stored in the tag/implant and then recovered, can be used on fishes released into open water (Fig. 6). Recovery of biologging tags remains a constraint, in particular for species that are not fished commercially or are under a fishing moratorium. A low recovery rate can make this method very costly, not only for the initial investment in tags but for the effort to implant them. Biologgers can collect and store both physiological variables (e.g. electrocardiogram, acceleration) and environmental parameters (e.g. pressure, temperature) that can be used to reconstruct migration pathways (Metcalfe and Arnold, 1997; Hunter *et al*., 2004), link behaviour to environmental conditions (Righton *et al*., 2001, 2010; Sims *et al*., 2003), characterize population structure (Metcalfe, 2006) and estimate energetic costs of different behaviours or interactions with humans (reviewed by Cooke *et al*. 2016). Pop-up tags that store data are also now widely used in studies on marine fishes, with the major advantage that data can be recovered via satellite. There are limitations to the size of fish that can carry the tags, and the tags can be expensive. Thus, most research has been on large and economically valuable pelagic or demersal fishes where, however, pop-ups have provided valuable knowledge for management and conservation (e.g. Block *et al*., 2011; Whitlock *et al*., 2015). In most cases, these tags are not ‘biologgers’; they store data only on environmental parameters, such as temperature and pressure.

An exciting development is the application of three-axis accelerometer tags (both in telemetry and biotelemetry/logger platforms) to monitor energy expenditure (Metcalfe *et al*., 2016a), activity and state. Movement is one of the four main bodily functions that incur energetic costs in animals. The energy expenditure is governed by muscle contractions and is typified by variable acceleration of the body (Gleiss *et al*., 2010), so records of the tri-axial acceleration of fishes should provide a useful proxy for activity-specific energy costs. Recent studies have correlated dynamic tri-axial body acceleration with rates of oxygen uptake in various aquatic species, including hammerhead sharks (*Sphyrna lewini*; Gleiss *et al*. 2010) and European sea bass (*Dicentrarchrus labrax*; Wright *et al*., 2014). Bi-axial and tri-axial acceleration, root mean square acceleration and acoustically transmitted acceleration data have also provided some exciting insights into fish behaviour and physiology (Clark *et al*., 2010; Wilson *et al*., 2013; Marras *et al*., 2015b). High-frequency accelerometry can be used to distinguish among various behaviours, such as feeding strikes and anti-predator escapes (Broell *et al*., 2013).

There are potential applications for biotelemetry, especially biologging, that can be highlighted for marine fish conservation research (Metcalfe *et al*., 2012). Tracking can improve our understanding of seasonal movements and space use and would be invaluable for evaluating the design and effectiveness of marine protected areas and to identify potential spawning aggregations. The addition of measurements of pressure, temperature and acceleration to the tracking devices can enable investigation of ontogenetic changes in the behaviour of pelagic species and evaluation of options for selective fishing strategies. Biotelemetric/logging data can also be combined with other assays, in particular of blood chemistry, to determine post-release survival of bycaptured (or sport-fished) animals. Environmental data collected by tracking or biotelemetric/logger tags can be used to define behavioural thresholds for critical habitat parameters, such as temperature (Neat and Righton, 2007; Righton *et al*., 2010), oxygen concentration (Prince *et al*., 2010) and salinity. This remains, therefore, a very exciting area of research and technological development.

## Making physiology relevant to decision-making

The value of mechanistic physiological information is currently not widely appreciated by resource managers and policymakers, not least because physiologists have not made a consistent effort to promote their science in this regard (Cooke and O'Connor, 2010; Horodysky *et al*., 2015, 2016; Patterson *et al*., 2016). There is a real opportunity to develop fish environmental physiology as a discipline, by contributing to conservation research (Cooke *et al*., 2013; Madliger *et al*., 2016). Physiology can reveal mechanisms, which can be used to explain ecological patterns, which may then support evidence-based predictions and management decisions (Cooke *et al*., 2013; Madliger *et al*., 2016). Physiological tools and knowledge have already contributed to conservation goals for marine fishes; for example, to the management of migrating Pacific salmon, to improving survival from bycatch in specific fisheries, or to reducing the impact of tourism on some natural fish populations (Madliger *et al*., 2016; Patterson *et al*., 2016). Physiology should also be able to inform policy decisions about the following: limiting mortalities from discards from many further fisheries; the design of marine protected zones; adaptation to global change; predicting potential for invasive species; and many other things.

In this Theme Section, Horodysky *et al*. (2016) and Patterson *et al*. (2016) provide thoughtful analyses of how physiological research and the research process relate to the needs of resource managers and their decision-making process. Physiology must contribute to a broader toolbox or conceptual framework within which policy operates. Although mechanistic insight can be very useful for managers and physiology can provide a component of this, it cannot be the only source of information; it must be considered alongside genetics, behavioural ecology, trophic webs, physical oceanography, and so on (Horodysky *et al*., 2015, 2016). Patterson *et al*. (2016) synthesize the reasons why physiological research on sockeye salmon (*Oncorhynchus nerka*) migration contributed successfully to management decisions in British Columbia (Canada). A main driver was an existing political motivation, based on observations of reduced salmon runs that seemed linked to rising river temperatures, which then funded targeted research; that is, there was a direct connection between a management problem and funding of physiologically based solutions. The collaboration was then successful because of accountability, legal clarity, effective institutional environments, good personal relationships and peer acceptance. Interactions between researchers and stake-holders were crucial, so that the people most affected by decisions were familiar with the research and so that personal relationships improved overall trust. Patterson *et al*. (2016) urge researchers to be aware of the need to provide confident predictions regarding future outcomes, which are tailored to specific management objectives; in particular, to be able to quantify uncertainty to the level desired by managers or other knowledge practitioners.

**Figure 7:**
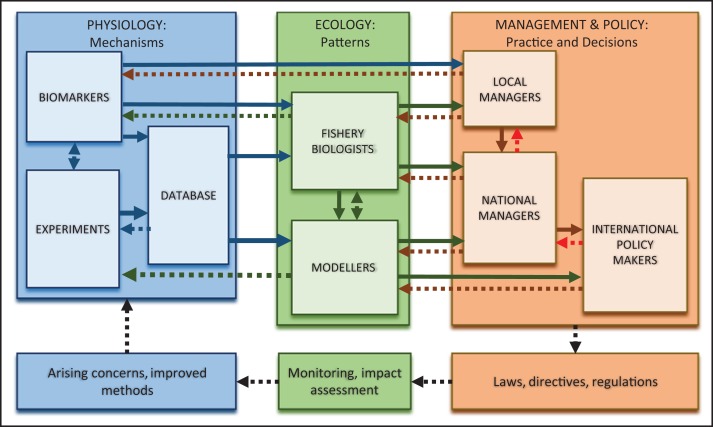
Flow diagram of how physiological information can inform management and/or policy decisions (continuous lines) for marine fishes, and how analysis of the information can be fed back to develop targeted research activities (dotted lines). Red dotted arrows show flow of policy decisions (in the European Union). Biomarker information can be used directly for local management (in particular, early warning and evaluation of ecological status). Physiological information can also influence national and international management/policy indirectly, by interactions with ecologists; for example, biomarkers of bycatch survival to inform fishery biologists, or physiological databases for use in modelling of population dynamics or effects of global change. The number of dotted lines feeding back to physiology reveal the many contributions that physiological research could make to adaptive management programmes, including large-scale and long-term research in response to, for example, EU or Intergovenmental Panel on Climate Change recommendations.

Fish physiologists generally lack contact with key policymakers and do not have direct information channels to attune and balance their research with policy decisions. Thus, a pervasive challenge is to integrate with other disciplines and scale up from physiology to decision-making. Fishery biologists, by the nature of their work, do have direct contacts and therefore represent an important link to policy for physiologists. Figure 7 is a flow diagram of how physiological research can inform management and policy decisions, and the feedbacks to research that can be used for adaptive management strategies (Williams, 2011). Monitoring of biomarkers, at immediate time scales, can provide advice for specific local conservation decisions; for example, to assess ecological status of coastal zones or for early warning of impacts of global change. This can feed back to elicit more focused biomarker research and monitoring at the local scale, but also feed forward to national and international monitoring; for example, in the context of the EU Water Framework Directive. As mentioned, however, another route to influence policy is through increased interactions with fishery biologists. Information about biomarkers of survival after discard can be provided to fishery biologists, in support of advice they might provide at a national level (Fig. 7). Information from field and laboratory physiology studies can populate databases, in order to then parameterize models to project ecological consequences at the level of populations, species and assemblages. These can influence wider-scale policy decisions, over longer time scales (Cooke *et al*., 2014). This flow of information may reveal knowledge gaps and longer-term policy priorities that, in turn, can feed back to drive more research; for example, in large international collaborative projects.

It is worthwhile to consider how detailed knowledge of the physiology, ecology and life history of marine fishes might be distilled for easy application in wide-scale decision-making on sensitivity of communities and assemblages to environmental change. Biological traits analysis has often been applied to assess the impact of environmental change on terrestrial and freshwater communities but, so far, has relatively few applications to marine fishes (Elleouet *et al*., 2014). Biological traits analysis holds promise because physiological, ecological and life-history trait data exist for many marine fish species, and an analysis of available information is the first step towards constructing a trait-based index of climate change sensitivity, in order to identify which aspects would be needed to develop the index as a tool. This would then be assessed against existing community-level data from observational studies, at sites that have been subject to recent and documented environmental change. Ideally, in the future, such functional sensitivity indices could be used to construct simple models of species extinctions and predict the likely impact of climate change on biodiversity and community function.

Initial attempts at bridging gaps among marine fish physiologists, ecologists, modellers and policymakers have been made, which bear reporting here. Roundtable discussions at a conference, funded by Action FA1004, were aimed at understanding some of the barriers to knowledge exchange between physiological and advisory processes, how to refine policy-management issues so they can be reflected better in conservation research, and whether fish physiologists have sufficiently considered the impact of their research on stakeholder and policy advice. A diversity of views was expressed, and the discussions are best summarized as the following general themes.

- The need for commonality of language. Dialogue is needed to achieve common understanding among physiologists, modellers and policy advisors. The simplest of terms can have a different meaning among scientists from different fields, and among stakeholders. This needs to be overcome without diminishing the autonomy of the various disciplines. A glossary of common terms, linked to the database of physiological information, would be useful.
- Temporal scales are often different for research and policy. Physiology may not be able to provide rapid advice to support a pressing policy decision. Fishery discards are a prime example, where policy changes resulted from societal pressure and not scientific understanding. Robust scientific underpinning would have required detailed and complex studies, achieved too slowly for policymakers. Such policy can, of course, then fund *post hoc* research to investigate physiological impacts of discarding and the likelihood of surviving it for the relevant species. A troubling example of temporal asynchrony is the lack of immediate concrete policy responses to evidence of profound effects on marine ecosystems of gradual ongoing climate change (Elliott *et al*., 2015).
- Don't give me the details, just the summary. Physiologists are interested in mechanistic detail, the responses of individuals and populations to changes in environmental conditions, typically of one particular model species. Such details often, however, run counter to effective stakeholder engagement and/or the advice needed for policy, which require synthesis of information into tangible effects. For example, the implications for fishing and fishermen, for future scenarios on fishing areas and species (in the context of climate change) or for the number and size of marine protected areas. Physiologists must learn to present their information in a holistic and understandable manner, including presentation to others in the scientific community, such as ecologists and modellers, who could translate, interpret and summarize the consequences for policy advice.
- Physiologists need to champion their cause. Physiologists must recognize that their research has impact, particularly through interaction with other related disciplines. It is not sufficient for physiologists to provide the data to parameterize models, then to dissociate themselves from the modelling outcomes. They must contribute to interpretation of results, in order to influence policy decisions. Physiologists need to understand the realms of policy work and policy decisions better, and the linkages from physiology to ecology then policy, in order to influence outcomes through co-production of knowledge and transdisciplinary research.

## Conclusions

There is much potential for physiological research to contribute to conservation of marine fish biodiversity and fisheries, which strengthens fish environmental physiology as a discipline. There is a clear need to increase the overall knowledge base about marine fish environmental physiology, especially tolerance thresholds for major environmental stressors and how such stressors affect performance within their tolerated range. Physiologists should explore avenues for international collaborative research, in order to avoid duplication of effort and cover as broad a range of species as possible. A particular application of such data would be to improve the reliability of models in order to gain a better understanding of what defines current fish distribution and abundance and, therefore, to increase confidence in projections of the effects of ongoing global change. Increased interaction with researchers using other tools, notably fishery biologists, ecologists and modellers, will provide a very fruitful avenue to increase the scope and impact of marine fish conservation physiology research and to make such research relevant to policy decisions.
